# Assessment of Intestinal Permeability and Inflammation Bio-Markers in Patients with Rheumatoid Arthritis

**DOI:** 10.3390/nu15102386

**Published:** 2023-05-19

**Authors:** Christina Heidt, Ulrike Kämmerer, Manfred Fobker, Andreas Rüffer, Thorsten Marquardt, Monika Reuss-Borst

**Affiliations:** 1 University of Muenster, D-48149 Muenster, Germany; 2 Department of General Pediatrics, Metabolic Diseases, University of Muenster, Albert-Schweitzer-Campus, D-48149 Muenster, Germany; marquat@uni-muenster.de; 3 Department of Obstetrics and Gynaecology, University Hospital of Wuerzburg, D-97080 Wuerzburg, Germany; u.kaemmerer@uni-wuerzburg.de; 4 Centre of Laboratory Medicine, University Hospital Muenster, D-48149 Muenster, Germany; manfred.fobker@ukmuenster.de; 5 Labor LS SE, D-97708 Bad Bocklet, Germany; andreas.rueffer@labor-ls.de; 6 Hescuro Center for Rehabilitation and Prevention Bad Bocklet, D-97708 Bad Bocklet, Germany; 7 Department of Nephrology and Rheumatology, Georg-August University of Goettingen, D-37075 Goettingen, Germany

**Keywords:** rheumatoid arthritis, zonulin, calprotectin, LPS, fecal short-chain fatty acids, fiber intake, intestinal permeability, intestinal inflammation

## Abstract

Increased intestinal permeability and inflammation, both fueled by dysbiosis, appear to contribute to rheumatoid arthritis (RA) pathogenesis. This single-center pilot study aimed to investigate zonulin, a marker of intestinal permeability, and calprotectin, a marker of intestinal inflammation, measured in serum and fecal samples of RA patients using commercially available kits. We also analyzed plasma lipopolysaccharide (LPS) levels, a marker of intestinal permeability and inflammation. Furthermore, univariate, and multivariate regression analyses were carried out to determine whether or not there were associations of zonulin and calprotectin with LPS, BMI, gender, age, RA-specific parameters, fiber intake, and short-chain fatty acids in the gut. Serum zonulin levels were more likely to be abnormal with a longer disease duration and fecal zonulin levels were inversely associated with age. A strong association between fecal and serum calprotectin and between fecal calprotectin and LPS were found in males, but not in females, independent of other biomarkers, suggesting that fecal calprotectin may be a more specific biomarker than serum calprotectin is of intestinal inflammation in RA. Since this was a proof-of-principle study without a healthy control group, further research is needed to validate fecal and serum zonulin as valid biomarkers of RA in comparison with other promising biomarkers.

## 1. Introduction

Rheumatoid arthritis (RA) is an incurable chronic inflammatory autoimmune disease that affects about 0.5–1% of the general population worldwide and is characterized by joint swelling, pain, the presence of autoantibodies (rheumatoid factor and anti-citrullinated protein antibodies (ACPA)), and subclinical signs of inflammation that result in aggressive synovitis and destruction of joint cartilage and bone [1,2]. The cause and pathogenesis of RA are still not completely understood, but are generally considered to be related to environmental and genetic factors [3]. Recent studies have demonstrated an alteration in intestinal microbiota in early and established RA [4,5]. Increased intestinal permeability and intestinal inflammation, both fueled by dysbiosis, have been suggested as factors that contribute to RA pathogenesis via a number of mechanisms, e.g., the activation of antigen-presenting cells and T-cells, and the alteration in the integrity of the intestinal epithelium [6,7].

Tajik et al. were the first who demonstrated in a collagen-induced arthritis model of how intestinal inflammation and a loss of permeability precede the onset of arthritis [8]. Additionally, Matei et al. confirmed such findings by showing a loss of intestinal integrity characterized by a reduced expression of tight junction protein 1, the translocation of bacterial products into serum and lymphoid organs, and increased lipopolysaccharide (LPS) levels before the development of arthritis [9].

The major function of the intestinal epithelial barrier is to prevent the passage of bacteria, other pathogens, and toxins from the lumen into the gut tissue and into systemic circulation [10]. Intestinal permeability is controlled by a fine balance of both adherence junctions and tight junctions (TJ) between epithelial cells, the latter being formed mainly by zonula occludens (ZO) proteins, claudins, and occludins, which anchor the transmembrane proteins to the actin cytoskeleton [11]. The interaction between the tight junctions and the actin cytoskeleton is fundamental to maintaining the tight junction structure, allowing the regulation of the paracellular pathway [12].

Assessing gut barrier function in humans is challenging and has been a matter of debate for many years [13]. One of the widely used methods to measure intestinal permeability is the lactulose and mannitol (L:M) test, in which, after a period of fasting, subjects are asked to drink a solution containing these two sugars. Urinary excretion of the sugars is then measured several hours after ingestion of the solute, and the lactulose/mannitol ratio (LMR) is calculated as an indicator of permeability [14]. A higher urinary LMR is believed to indicate higher small intestine permeability [14]. Such functional tests can provide good diagnostic accuracy when implemented by an experienced examiner and offer some insight into gut permeability but are limited by the lack of standardization, the length of time required to conduct the tests, and by the inability to perform the tests retrospectively. The test results can also be compromised by concomitant intestinal disease or non-steroidal anti-inflammatory drug (NSAID) intake [13,15,16,17].

To simplify the assessment of gut permeability and inflammation, studies investigating several biomarkers, such as zonulin, calprotectin, and lipopolysaccharide (LPS), have been proposed [18,19].

Zonulin, a protein produced by small intestine epithelium, is a potent regulator of endothelial and epithelial tight junctions [20,21]. Increased serum zonulin concentrations are related to changes in tight junction competency, loss of the intestinal barrier, dysbiosis, and inflammation in new-onset and established-RA patients [8,22].

Calprotectin is secreted predominantly from stimulated neutrophils [23]. It has a bactericidal effect and promotes inflammatory responses [23]. Higher calprotectin levels have been found in synovial fluid, plasma, and serum from RA patients [24,25,26]. Calprotectin levels are not affected by age or gender, and there is a growing body of evidence to support its greater accuracy in stratifying disease activity than CRP and ESR [25,26,27]. Serological calprotectin is currently being studied as a promising biomarker of RA disease activity [28,29].

Lipopolysaccharides (LPSs) are complex amphiphilic molecules, which are released from Gram-negative bacterial membranes via shedding or through bacterial lysis. LPS is a widely studied inflammatory molecule [30]. In pathologic conditions, the intestinal tissue and circulating LPS levels are markedly elevated and play an important role in mediating inflammatory response [31].

Since the gut is not the only source of circulating zonulin and calprotectin, data on the association of serum levels and intestinal are ambiguous [32]. Moreover, little is known about the correlation between blood and fecal zonulin and calprotectin levels or which may be the more valuable diagnostic and prognostic biomarker of RA.

The objectives of this study were to investigate which of the established biomarkers of impaired intestinal damage and permeability (zonulin, LPS) and intestinal inflammation (calprotectin) correlate with pathogenic findings in RA, apart from microbiome analysis, to compare, for the first time, fecal and blood levels of zonulin and calprotectin in patients with RA and determine the diagnostic value of each of these analyses. Previous studies have used either fecal biomarkers or serum biomarkers but have not compared both fecal and serum biomarkers within the same study. Furthermore, we aimed to assess whether or not there are associations of zonulin and calprotectin with LPS, BMI, gender, age, smoking status, RA-specific and other biochemical parameters, fiber intake, and the presence of short-chain fatty acids in the gut.

## 2. Materials and Methods

### 2.1. Study Design and Patients

The study was a single-center, pilot study of RA patients, who were under regular follow-up at Rheumatology Outpatient Practice in Bad Bocklet, Germany. This study was conducted from August 2021 to October 2022.

The inclusion criteria were adult patients (>18–80 years), with no evidence of metabolic disease other than obesity, and a diagnosis of rheumatoid arthritis fulfilling the RA classification criteria of the American College of Rheumatology/European League Against Rheumatism (ACR/EULAR) [33]. Since the few smokers in the group (Table 1) were only occasional to moderate smokers, we did not exclude smokers from the trial. Exclusion criteria from the study were BMI > 45 kg/m^2^, antibiotic, prebiotic, and probiotic therapy up to 3 months before the study, vegan diet, ketogenic diet, type 2 diabetes, cardiovascular disease, neurological and psychiatric disorders, and inflammatory bowel disease. Since a higher fat mass is prevalent in RA, we did not exclude patients with obesity, but we included otherwise healthy obese RA patients without inflammatory disorders, e.g., type 2 diabetes [34]. Secondly, since elevated zonulin has been associated with various other chronic inflammatory diseases, we excluded patients with such a history [35]. We included patients on a standard and/or vegetarian diet and excluded patients on a vegan diet, since, across all studies, average fiber intake is the highest in vegans (44 g/d), followed by vegetarians (28 g/d), and is the lowest in people on a common diet (21 g/d) [36]. We excluded patients receiving antibiotic, prebiotic, and probiotic therapy since such treatments may alter the microbiota and might affect systemic inflammation and the biomarkers of the intestinal barrier [37]. Additionally, patients on a ketogenic diet were excluded due to the high fat intake and production of ketone bodies in the liver [38]. Several studies have shown that a high fat diet alters gut microbiota and intestinal permeability [39,40]. Additionally, ketone bodies alter the human gut microbiota with downstream consequences for immune cells and associated decreases in intestinal Th17 cell levels, which are increased in RA patients [40].

The study was approved by the Bavarian Ethics Committee (approval number 21020) and was registered at the German Registry of Clinical Trials (DRKS00025413). All patients provided their informed written consent.

Sixty-one (61) consecutive adult patients were included in this study. Patient characteristics including gender, age, BMI, smoking status, RA-specific and biochemical data, treatment, and nutrition are provided in Table 1.

### 2.2. Fecal Sample Processing and Analysis

The whole stool specimens were collected by the patients (in accordance with the lab instructions) using a disposable plastic bucket-type device which avoids toilet water artifacts and simplifies laboratory sampling. The stool specimens were brought immediately to the laboratory (Labor LS/Enterosan, Bad Bocklet-Großenbrach, Germany) and analyzed for SCFAs (gas chromatograph GC 3900, Varian, Palo Alto, CA, USA), zonulin (ELISA kit, Immundiagnostik AG, Bensheim, Germany), and calprotectin (ELISA kit, Immundiagnostik AG, Bensheim, Germany).

In terms of *SCFAs*, each specimen was tested for concentrations of butyrate, acetate, propionate, iso-valerate and iso-butyrate via gas chromatography. Fecal samples were suspended in an isotonic NaCl solution. After homogenization and centrifugation, the supernatants were spiked with methacrylic acid and mixed with NaCl and 75% H_2_SO_4_. Diethyl ether was added, and the mixtures were then shaken with an overhead shaker (Reax 2, Heidolph, Schwabach, Germany) for 15 min. Thereafter, the mixtures were frozen at −25 °C for 6 h. The organic supernatants were analyzed for the presence of SCFA using a gas chromatograph, GC 3900 (Varian, USA), fitted with a column, BP-21 (SGE Analytical Science, USA), and a flame ionization detector. The results of the test samples were calculated against methacrylic acid as the internal standard and were expressed in µmol/g; the reference value for SCFA is ≥14 µmol/g, and the reference value for butyrate is ≥2.5 µmol/g, both values corresponding to those indicated by the lab. Fecal samples were available from 60/61 patients for SCFA analysis and 59/61 patients for butyrate analysis. In total, 2/61 patients did not provide enough stool samples for these analyses.

*Fecal calprotectin and fecal zonulin* were tested using CE-marked commercial ELISA (enzyme-linked immunosorbent assay) kits in accordance with the manufacturer’s instructions (ELISA kits from Immundiagnostik AG, Bensheim, Germany). For these analyses, the amount of fecal samples was sufficient for all 61 patients. Fecal samples were suspended in a diluent buffer. After homogenization and centrifugation, the supernatants were transferred onto microplates, each coated with antibodies specific to the respective inflammatory markers. Anti-zonulin or anti-calprotectin antibodies, each conjugated with peroxidase, were used for development. In addition to the fecal samples, standards as well as positive and negative controls were tested for all parameters. For each well, the optical density was measured at 450 nm with a microplate ELISA reader (Dynex, Denkendorf, Germany). The results of the test samples were calculated from the standard curve and were expressed in ng/g (zonulin) or µg/g (calprotectin); the reference value for calprotectin is <50 µg/g, and the reference value for zonulin is <61 ng/g, both values corresponding to those indicated by the lab.

### 2.3. Blood Sample Processing and Analysis

Blood samples were collected after at least 8 h of fasting from all patients. Serum tubes (BD Vacutainer, Franklin Lakes, NJ, USA, 5.0 mL) were centrifuged at 700× *g* for 10 min at room temperature, while aliquots of sera or plasma, were transferred into 1.5 mL microtubes (Eppendorf, Hamburg, Germany) and stored at −80 °C until the assay was carried out. Analyses were conducted with a Cobas 4000 analyzer (Roche Diagnostics, Mannheim, Germany).

Serum calprotectin and serum zonulin were evaluated using CE-marked competitive enzyme-linked immunosorbent assay (ELISA) kits from Immunodiagnostik AG (Bensheim, Germany) in accordance with the manufacture’s instruction. Zonulin concentration in serum was presented in ng/mL. Based on the manufacturer’s studies of apparently healthy persons, a mean value of 34 ng/mL (±14 ng/mL) was estimated as the reference range. Calprotectin in serum was presented in µg/mL, and a value lower than 3 µg/mL was considered as normal, according to the manufacturer. Serum samples were collected from 61/61 patients for zonulin and calprotectin analysis.

Plasma LPS was evaluated by a kit obtained from Cloud-Clone Corp. (Katy, TX, USA). The accompanying protocol was followed with no deviations: using a 96-well stripe plate pre-equilibrated to 37 °C, wells were first filled with 100 μL of sample or standard. Then, 50 μL of the prepared detection reagent, reagent A, was added immediately, shaken, and incubated for 1 h at 37 °C. To this, 100 μL of detection reagent B was added and the plate was incubated at 37 °C for 30 min. Next, 90 μL of a pre-equilibrated (to 37 °C) substrate solution was added to each well, and the plate was incubated at 37 °C for another 15 min. Finally, 50 μL of a stop solution was added to each well, and the plate was read at 450 nm. The normal plasma concentration had to be below 0.05 ± 0.01 ng/mL [41]. Due to constraints imposed by the COVID-19 pandemic, plasma samples could only be collected from 30/61 patients for LPS analysis.

### 2.4. Fiber Intake Analysis

A 3-day estimated dietary record was collected from 61/61 patients and analyzed as previously described [42]. Dietary intake was quantified and analyzed by a nation-customized nutrition database (PRODI^®^ 6 expert, Nutri-Science GmbH, Freiburg, Germany).

### 2.5. Statistical Analysis

Statistical analyses were performed using GraphPad Prism, version 9.5.1 (GraphPad, La Jolla, CA, USA), and the R statistical programming language. In order to describe the distribution of biomarkers, location and spread were reported as medians and interquartile range. Frequencies of abnormal values, categorical variables of fecal and blood biomarkers related to RA and differences in study parameters between females and males were calculated using Fisher’s exact test. The level of statistical significance was set to *p* ≤ 0.05. The normality of residuals was examined using qq-plots and histograms. Linear regression and multivariate linear regression analysis were carried out to determine the associations between multiple variables using the statistical programming language R, version 4.2.1 (Package: finalfit). In order to describe the expression patterns using a heatmap (Package: pheatmap), log-transformed values were normalized via quantile normalization (Package: preprocessCore) [43]. For heatmap clusters, the Euclidean distance with Ward’s clustering method was used.

## 3. Results

### 3.1. Patient Characteristic

A total of 61 patients were enrolled in the study, comprising 20/61 (33%) males and 41/61 (67%) females. The median age was 65 (58–71) years for females and 57 (53.3–61.5) years for males, and the median disease duration was 3 (1–11.5) years for females and 0.8 (0.5–3.9) years for males. In this sample, 6/61 (9.8%) were current smokers (3 female and 3 male) and 29/61 (47.5%) had never smoked. Females had a median BMI of 25.7 (22.6–30.81) kg/m^2^ and 13/41 of them were obese (BMI > 30 kg/m^2^). Males had a median BMI of 27.8 (25.7–30.3) kg/m^2^ and 6/20 of them were obese (Supplementary Table S1).

Only 2 of the female patients were vegetarians, and 59/61 were on a standard diet. Detailed demographic and clinical data of the enrolled RA patients are presented in Table 1.

### 3.2. Fecal Biomarker Concentrations and Fiber Intake

#### 3.2.1. Fecal Zonulin

The median zonulin concentration in fecal samples was 297 (226–413) ng/g and 98% over the reference value from the manufacturer. There was no significant difference in zonulin fecal levels between females and males (Table 2).

#### 3.2.2. Fecal Calprotectin

The median calprotectin concentration was 36.1 (19.2–94) µg/g. A total of 17/41 females and 6/20 males had higher concentrations above the reference value of <50 µg/g. There was no significant difference in calprotectin fecal levels between females and males (Table 2).

#### 3.2.3. Fecal Total Short-Chain Fatty Acids (SCFAs), Butyrate Concentrations and Fiber Intake

Concentrations of SCFAs and butyrate are shown in Table 2. Two out of sixty patients had abnormal levels of total SCFAs according to reference value from the lab. Fifteen out of fifty-nine patients had levels lower than the reference value provided by the lab. No significant differences in total SCFAs and butyrate levels between females and males were found.

SCFAs are metabolites of bacterial fiber fermentation in the gut. The median fiber intake was 15.26 (10.74–20.26) g/day (Table 1). There was no significant difference in fiber intake between female and male patients (*p* = 0.8).

### 3.3. Blood Biomarkers Concentrations

#### 3.3.1. Serum Zonulin

The median zonulin concentration was 32 (28–42) ng/mL. A total of 16/41 females and 7/20 males had elevated levels that were above the manufacturer’s reference value. No significant difference in zonulin serum levels between females and males were found (Table 2).

#### 3.3.2. Serum Calprotectin

The median calprotectin concentration was 2 (1.3–2.9) µg/mL. A total of 10/41 females and 4/20 males had elevated levels that were above the reference value provided by the manufacturer. There was no significant difference in serum calprotectin levels between the sexes (Table 2).

#### 3.3.3. Plasma LPS

Plasma LPS concentrations are presented in Table 2. Abnormal LPS levels were significantly higher in women compared to men (*p* = 0.018).

### 3.4. Relationship between Fecal and Blood Biomarkers, SCFAs, Fiber Intake, Gender, Smoking Status and Obesity

**Figure 1:**
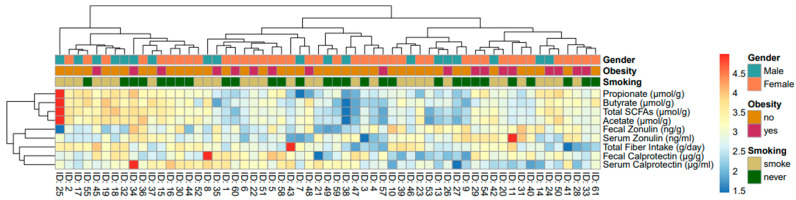
Heatmap (*n* = 59) including gender, smoking status and obesity.

Figure 1 shows a heatmap of fecal and serum biomarkers, SCFA and fiber intake, gender, smoking, and obesity. The colors (red = most frequent; dark blue = least frequent) show the relationship between biomarkers, SCFAs, and fiber intake of each patient. The heatmap appears to outline a stronger relationship between SCFAs and fecal zonulin and a weak relationship between the former and serum zonulin. The heatmap also shows that fiber intake has a weak relationship with the presence of total SCFAs in the gut. There is no relationship between gender, obesity, and smoking with the biomarkers.

### 3.5. Frequencies of Abnormal Values of Fecal and Blood Biomarkers Related to RA

Table 3 lists the significant and trend frequencies of abnormal values of fecal and blood biomarkers. Non-significant frequencies of abnormal concentrations of fecal and blood markers related to BMI, smoking status, disease duration, SDAI, ACPA, and drug treatment are presented in the Supplementary Materials (Table S2).

Abnormal serum zonulin levels were significantly more common in RA patients with a positive rheumatoid factor (RF) and lower CRP concentrations. Abnormal fecal calprotectin levels were significantly more common in RA patients treated with glucocorticoids. There was also a weak trend towards abnormal LPS levels in non-obese RA patients (*p* = 0.095), and abnormal fecal calprotectin levels in patients treated with TNF inhibitors (*p* = 0.084) (Table 3).

### 3.6. Associations between Serum and Fecal Zonulin, Serum and Fecal Calprotectin, and LPS and RA-Specific Confounders—Univariate Linear Regression

**Figure 2:**
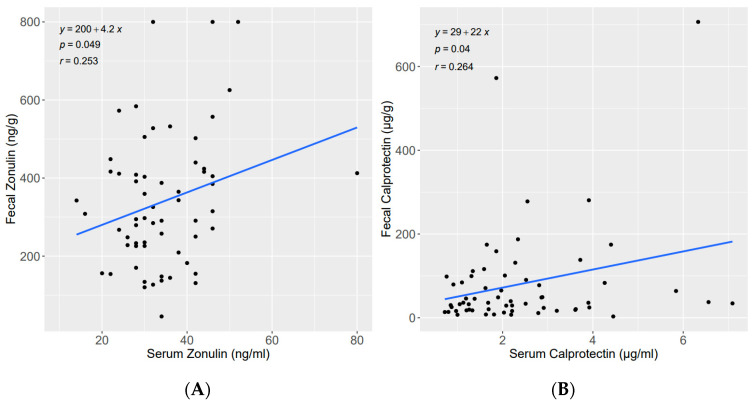
Linear regression (**A**) between fecal zonulin (ng/g) and serum zonulin (ng/mL); (**B**) between fecal calprotectin (µg/g) and serum calprotectin (µg/mL).

In the univariate linear regression analysis, both fecal zonulin and calprotectin were significantly associated with serum zonulin and calprotectin (Figure 2).

There were no statistically significant associations between either fecal zonulin or calprotectin with plasma LPS. Furthermore, serum zonulin and calprotectin showed no significant associations with plasma LPS (Supplementary Materials Figures S1 and S2).

**Figure 3:**
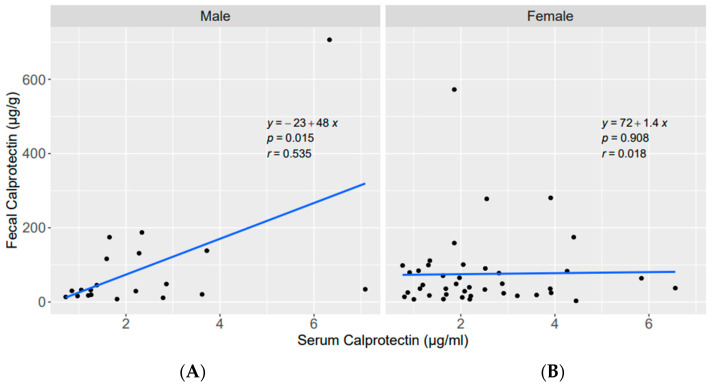
Linear regression between fecal calprotectin (µg/g) and serum calprotectin (µg/mL) (**A**) in males; (**B**) in females.

**Figure 4:**
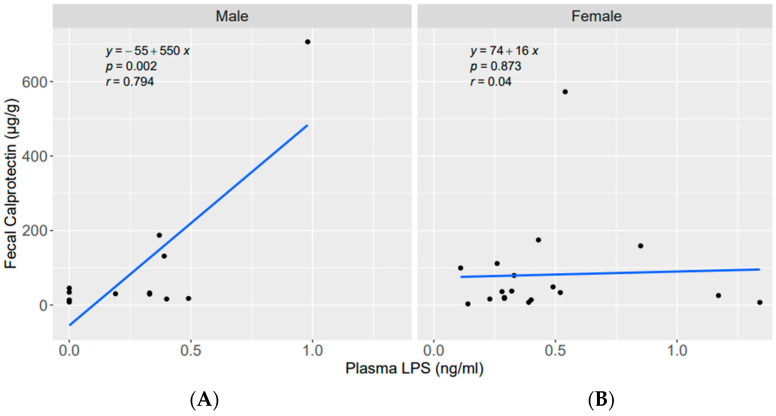
Linear regression between fecal calprotectin (µg/g) and plasma LPS (ng/mL). (**A**) in males; (**B**) in females.

Additionally, we compared associations between zonulin, calprotectin and plasma LPS in females and males. Interestingly, no significant associations could be detected for these biomarkers in females (Supplementary Materials Figures S3–S6). While significant associations of fecal calprotectin with serum calprotectin and of fecal calprotectin with plasma LPS were found in males (Figure 3 and Figure 4)**,** no correlation was seen in females.

### 3.7. Associations between Blood and Fecal Biomarkers and RA-Specific Confounders—Multivariate Linear Regression

We performed multiple linear regression analyses with the biomarkers as dependent variables and plasma LPS, gender, age, RA-specific and other biochemical parameters, fiber intake, and presence of short-chain fatty acids in the gut as independent variables. Serum zonulin levels were strongly associated with fecal zonulin levels and were also affected by disease duration, with no significant effect of gender, age, BMI, SDAI, and LPS. Serum calprotectin levels were strongly associated with fecal calprotectin, without the effects of gender, age, BMI, SDAI, and LPS. Both fecal zonulin and calprotectin levels were associated with serum zonulin and calprotectin, yet this association was not statistically significant after multivariate linear analysis. There was a significant inverse association between fecal zonulin levels and age. The univariate linear regression model showed that the amount of fecal zonulin was by trend inversely associated with fecal butyrate (Table 4). Details of further non-significant results are presented in Supplementary Materials (Table S3).

## 4. Discussion

It is common in inflammatory bowel disease (IBD) and in RA research to use commercially available kits to assess zonulin, a marker of intestinal permeability, or calprotectin, a marker of intestinal inflammation, in blood or stool [8,23,44,45,46,47,48,49,50]. However, there are insufficient data regarding which (fecal or serum) may be the more valuable diagnostic and prognostic biomarker for RA. To our knowledge, this is the first study that compares fecal and serum levels of zonulin and calprotectin in RA patients using commercially available ELISA kits. The reported markers are not disease-specific but corroborate the hypotheses of intestinal dysbiosis as a contributing factor in RA [8,9,22,51].

In this study, fecal zonulin was markedly increased in nearly (98%) of all patients (100% of females, and 95% of males) according to the manufacturer’s reference value of greater than 61 ng/mL indicating increased intestinal permeability [45,52]. However, it should be noted that some authors have established their own internal upper reference limits for fecal zonulin (<30 ng/mL) using the same commercial ELISA assays, referring to previous studies and their own experience [52,53,54,55]. Given that an elevated level of the fecal protein zonulin reflects high gut permeability, these findings provide evidence that increased gut permeability is already present in RA patients at the earliest stages [8,22]. In addition, fecal zonulin levels were strongly associated with serum zonulin levels and were also inversely associated with age. It is important to note that the reference values provided by the manufacturer are based on a small number of healthy subjects and apply to all ages. [56]. Therefore, the results of this study indicate the need for further studies on the methodological side to clarify the ideal reference values to further strengthen the exact associations between fecal zonulin levels as biomarkers and in people with RA.

Other important findings in this study were that elevated serum zonulin concentrations were significantly more common in patients with a positive rheumatoid factor status, that levels of serum zonulin were significantly associated with fecal zonulin, and that abnormal serum zonulin was significantly associated with disease duration, as supported by univariate and multivariate analysis. This last finding concurs with that of a previous study showing that abnormal serum zonulin concentrations are common in new-onset RA patients and that abnormal serum zonulin levels appear more frequently and tend to be higher in established-RA patients [8]. However, it should be noted that Scheffler et al. reported that the ELISA kit used in the present study does not detect serum zonulin directly, but rather concentrations of properdin, which could potentially affect the results [57,58,59]. Zonulin is secreted not only from enterocytes but also, reportedly, adipose tissue, brain, heart, immune cells, liver, lungs, kidney, and skin [32,60]. Thus, serum zonulin levels are not specific to intestinal secretion and therefore, fecal zonulin may be more specific as a marker of intestinal permeability since it appears to leak from the intestinal barrier into the lumen [32]. This issue may not be relevant if these ELISA kits correlate strongly with functional gut permeability as assessed via established tests [13]. At present, this is the best method for measuring zonulin levels in human samples and is widely used for measuring intestinal permeability [61].

Environmental factors such as diet may have an important role in regulating intestinal integrity [61,62]. A study that targeted intestinal permeability by treating mice with butyrate found that a reduction in the severity of the observed arthritis was due to the improvement of intestinal permeability and was associated with a reduction in serum zonulin concentrations [8]. Short-chain fatty acids (SCFAs) including butyrate, acetate, and propionate are the main end products of bacterial fiber fermentation in the gut [63]. The recent literature shows that a high-fiber diet increases SCFA levels and decreases inflammatory burden in patients with established RA [64,65,66]. This study showed inconsistent associations between serum zonulin and fiber intake, SCFAs, and, specifically, butyrate. Only a weak, inverse association was observed for fecal zonulin and butyrate, and the association was even weaker for fecal zonulin and total SCFAs. The absence of stronger associations between serum and fecal zonulin and SCFAs might be explained by our results showing that only 3% of patients had abnormal SCFAs and just 25% had abnormal butyrate values. Our results differ from those obtained by Abendroth et al. who reported lower SCFA levels (using the same methodology and lab) in patients consuming a Mediterranean diet, which is considered to be high in fiber [67]. These findings are debatable since the mean dietary fiber intake of the people in our study was only 15.26 g (10.74–20.26) per day. It is noteworthy that fiber consumption in Germany is generally low and the recommended daily amount of 30 g of dietary fiber per day is not commonly reached [68]. Therefore, inadequate fiber intake might explain the lack of a significant association. The normal levels of SCFAs despite the lower average fiber intake in our RA patients could be also explained by the presence of adequate numbers of gut microbes that produce SCFAs through fermentation [69]. Therefore, more interventional studies including microbiome analysis are needed to clarify a possible link between zonulin and fiber intake, as well as the quality of the fiber source since soluble fiber tends to increase SCFA levels more than insoluble fiber does [70].

Higher zonulin levels are associated with metabolic conditions, e.g., larger waist circumference, higher fasting glucose levels, and increased risk of overweight, obesity, and hyperlipidemia [71,72]. It is known that zonulin concentrations vary with smoking [45,73]. This study found no associations between biomarkers and other environmental factors such as smoking, BMI, or obesity. This is possibly because the study sample did not have enough strength to show this difference, as only 6/61 were smokers and 19/61 were obese (BMI > 30 kg/m^2^). In addition, probiotics (live microorganisms) and synbiotics (containing probiotic strains and prebiotics) have been found to modulate the epithelial barrier by reducing serum levels of zonulin [74]. Several studies have been conducted to investigate the impacts of probiotics on serum or fecal levels of zonulin [75]. However, the results are inconsistent or inconclusive [75]. Therefore, we have excluded these confounders. Additionally, certain pathogenic gut microbes appear to induce the release of zonulin from the gut, suggesting a mechanistic link between alterations in the gut microbiota and gut barrier function [76]. Future studies should therefore investigate whether or not there is an association between these markers and the microbiota.

In contrast to fecal zonulin, calprotectin levels in stool and blood were elevated in 38% of patients (according to the reference values of the manufacturer), without associated gastrointestinal symptoms, since patients identified with these symptoms at screening were excluded. The higher fecal calprotectin level suggests that these RA patients may have had a subclinical gastrointestinal pathology, based on the manufacturer’s reference value of 50 µg/g. However, Roon et al. reported that using a cut-off of 100 µg/g in patients with IBD could provide greater diagnostic efficiency and precision, and just a few patients (13/61) in this study were above that value [77]. These results suggest setting a different cut-off point for fecal calprotectin for RA patients than for healthy adults.

Calprotectin is a heterodimer formed by two proteins, S100A8 and S100A9, which are the most up-regulated proteins in rheumatoid arthritis [47]. Calprotectin can be measured in serum, plasma, and feces and exhibits potency as an inflammatory non-systemic biomarker [78]. Increased serum concentrations of calprotectin have been found in RA patients in contrast to healthy controls [78]. A meta-analysis shows that blood calprotectin relates to disease risk, inflammation, and disease activity in RA patients [79]. Other studies have shown that elevated levels of calprotectin in serum are associated with positive anti-cyclic citrullinated peptide (anti-CCP), positive rheumatoid factor (RF), and disease activity parameters such as SDAI, Disease Activity Score 28 (DAS28) and CRP [79,80,81]. Contrary to these studies, the results of the study herein observed no association between serum or fecal calprotectin with CRP and SDAI. An explanation for the lack of association might be that patients with other disorders characterized by inflammation were excluded.

Probably the most interesting observation in this study was that abnormal fecal calprotectin concentrations were significantly more common in RA patients treated with glucocorticoids than in untreated patients, but no significance was found for serum calprotectin and glucocorticoid treatment. Some studies have reported that effective corticosteroid treatment decreases calprotectin serum levels in autoimmune disease, but an unexpected effect of corticoids of increasing calprotectin in serum has been observed in other studies [47,81,82]. There is evidence that corticosteroids can induce the expression of S100A8 in monocytes, dendritic cells, and synovial macrophages in RA [83]. An increase in serum calprotectin was also reported by Klingberg et al. along with a significant increase in white blood cells after corticoid administration [81]. Corticosteroids might therefore promote the expression of calprotectin, suggesting a possible link between greater intestinal inflammation and long-term corticosteroid treatment, which may have been the case in our long-treated patients [47]. This question could be addressed in future studies using larger patient cohorts that also search for associations of fecal and serum biomarkers with disease severity and medication use [9]. Higher calprotectin levels have been reported in the synovial fluid of people with RA compared to those with types of inflammatory arthritis and bone-related disorders [24,84]. However, the measurement of synovial calprotectin was beyond the scope of this pilot study. Future studies are needed to determine whether or not there are specific associations between blood, fecal, and synovial calprotectin and disease severity.

Ultrasound is a diagnostic technique widely used in rheumatology to assess joint inflammation with greater sensitivity [85]. Serum calprotectin has shown the strongest correlation with ultrasound [86,87]. However, the value of serum calprotectin as a biomarker of treatment response and flare-up after tapering still requires larger, standardized studies that include ultrasound to provide further evidence of specificity [23].

In the present study, a strong association between the levels of calprotectin in feces and serum was found, which according to our data, are independent of BMI, age, and RA-specific and other biochemical parameters. The same results were obtained in males, but not in females. To the best of our knowledge, this finding has not been published previously.

To add to the evidence on intestinal barrier dysfunction, we also analyzed plasma lipopolysaccharide (LPS) levels [88]. LPS levels were studied in place of the usual lactulose/mannitol excretion ratio.

LPS, a structural component of Gram-negative bacteria, is a known pyrogenic substance and is often used to promote the development of arthritis in animal experiments [89]. Elevated serum LPS levels caused by LPS absorption from the gut to elsewhere in the body are more frequently observed in patients with RA than in healthy controls, indicating that gastrointestinal barrier damage with LPS translocation into the bloodstream may play a role in the progression of RA by promoting inflammation, which is pivotal in RA [9,30]. In this study, 87% of patients showed elevated plasma LPS levels, which were significantly more common in females than in males. In addition, a significant association (supported by multivariate analysis) was found between fecal calprotectin and plasma LPS (in the total study population and in males), but not between serum calprotectin and LPS, which showed an inverse association. It is known that LPS can promote the expression of fecal calprotectin as a direct consequence of intestinal inflammation [30,90]. It has been shown that LPS produced by *E. coli* also increases the levels of fecal calprotectin [91]. The present study did not adjust for microbiome analysis, which is a limitation.

Since the practical use and limitations of biomarkers are currently under extensive discussion, this proof-of-principle pilot study serves as a good starting point for further studies that could focus on improving the performance and usefulness of potential biomarkers such as zonulin and calprotectin as diagnostic tests for patients with RA. However, the results obtained in this study should be taken with caution due to further limitations. First, the sample size of patients (males and females) was small, and the population was quite heterogeneous since some patients had longstanding disease, some patients were in remission, and others showed low to moderate disease activity, and, in addition, the patients were using a variety of medications, which could explain why we did not find a significant correlation between the biomarkers and RA-specific markers, apart from between serum zonulin and disease duration. Additionally, this study had no healthy control group. It is also important to mention that current commercial ELISA assays for zonulin identify several structurally similar proteins (haptoglobin) rather than zonulin specifically [58]. It is, consequently, still unclear what the commercially available zonulin tests detect, even if the values obviously correlate with intestinal permeability [57,58]. For these reasons, we urge for caution in relying on the indirect measurement of zonulin as the only marker of intestinal barrier integrity. The commercial ELISA detection methodology might be improved with development of specific monoclonal capture and detection for zonulin and haptoglobin in future studies. A further limitation of this study is that we did not determine the lactulose/mannitol excretion ratio, since the test requires prolonged fasting, and the periodic collection of urine, which is very time-consuming, and was not feasible due to constraints imposed by the COVID-19 pandemic during the timeframe of this study.

## 5. Conclusions

*What is known?*

- Zonulin, a biomarker of intestinal permeability, and calprotectin, a biomarker of intestinal inflammation, are elevated in serum and feces in patients with RA.
- The elevated serum and fecal zonulin levels suggest that increased gut permeability is present in RA.
- Plasma LPS, a marker of intestinal permeability and inflammation, is also elevated in RA patients.

*What is new?*

- A strong association between serum and fecal zonulin was found in patients with RA.
- Serum zonulin levels were more likely to be abnormal with a longer disease duration and fecal zonulin levels were affected inversely to age.
- A strong association between fecal and serum calprotectin and between LPS and fecal calprotectin were found in males, but not in females, independent of other biomarkers.
- Abnormal fecal calprotectin concentrations were significantly more common in RA patients treated with glucocorticoids than in untreated patients.
- Fecal calprotectin appears to be a more promising marker of intestinal inflammation than serum calprotectin does, which showed no association with LPS.

Since this was a proof-of-principle pilot study, further research that includes a healthy control group is necessary. Further studies should also focus on the comparison of biomarker analysis using commercially available kits with other testing methods, such the dual-sugar test and ultrasound, to ensure that the kits are appropriate for the detection and monitoring of increased gut permeability and inflammation in RA patients in clinical practice.

## Figures and Tables

**Table 1:** Patient characteristics (*n* = 61).

Demographic Data, Unit	Indexes	All	Female	Male
Gender	*n* (%)	61 (100)	41 (67)	20 (33)
Age, years	Median (IQR)	63 (56–70.5)	65 (58–71)	57 (53.3–61.5)
BMI, kg/m^2^	Median (IQR)	26.3 (23.5–30.9)	25.7 (22.6–30.81)	27.8 (25.7–30.3)
Smoking status				
Current	*n* (%)	6 (9.8)	3 (7.3)	3 (15)
Former	*n* (%)	26 (42.6)	16 (39)	10 (50)
Never	*n* (%)	29 (47.5)	22 (53.7)	7 (35)
**RA-specific data, unit**				
Disease duration, years	Median (IQR)	2.5 (0.6–7.5)	3 (1.0–11.5)	0.8 (0.5–3.9)
SDAI, units	Median (IQR)	11.08 (6.36–18.09)	11.9 (6.1–20.1)	10.2 (7.1–21.3)
CRP, mg/dL	Median (IQR)	0.19 (0.08–0.52)	0.2 (0.1–0.5)	0.2 (0.1–0.4)
Rheumatoid Factor IgM, positive	*n* (%)	21 (34.4)	15 (37)	6 (30)
Anti-CCP-IGG antibody, positive	*n* (%)	17 (27.9)	13 (32)	4 (20)
**Anti-rheumatic treatment**				
Methotrexate	*n* (%)	19 (31.1)	14 (34)	5 (25)
Other conventional (cs) DMARDs	*n* (%)	7 (11.5)	6 (15)	1 (5)
Targeted synthesized (ts) DMARDs	*n* (%)	3 (4.9)	2 (5)	1 (5)
Biologicals	*n* (%)	13 (21.3)	9 (22)	4 (20)
Glucocorticoids	*n* (%)	16 (26.2)	10 (24)	6 (30)
**Biochemical data, unit**				
Total cholesterol, mmol/L	Median (IQR)	6.01 (5.1–6.6)	6.08 (5.22–6.39)	5.34 (4.7–6.6)
Fasting triglycerides, mmol/L	Median (IQR)	1.27 (1.03–2.10)	1.28 (1.03–2.0)	1.37 (1.09–2.19)
Non-HDL cholesterol, mmol/L	Median (IQR)	4.21 (3.33–4.81)	4.19 (3.41–4.73)	3.96 (3.25–5.0)
**Nutrition intake data, unit**				
Energy, kcal/day	Median (IQR)	1509 (1281–1863)	1557 (1193–1806)	1493 (1350–1932)
Total fiber, g/day	Median (IQR)	15.26 (10.74–20.26)	15.3 (11.2–19.6)	15.6 (10.8–21)

BMI, body mass index; SDAI, simple disease activity index; CRP, C-reactive protein; CCP: cyclic citrullinated peptide; DMARDs, disease-modifying anti-rheumatic drugs.

**Table 2:** Values of fecal and blood biomarkers.

Biomarkers, Unit	Indexes	All (N = 61)	Female (N = 41)	Male (N = 20)	*p*-Value
Fecal zonulin, ng/g	Median (IQR)	297 (226–413)	326 (248–417)	285 (166–406)	0.33
Reference value according to the lab	ng/g	<61	<61	<61	
Abnormal values	*n*/N (%)	60/61 (98)	41/41(100)	19/20 (95)	
Fecal calprotectin, µg/g	Median (IQR)	36.1 (19.2–94)	40 (20–84)	32 (19–120)	0.42
Reference value according to the lab	µg/g	<50	<50	<50	
Abnormal values	*n*/N (%)	23/61 (38)	17/41 (42)	6/20 (30)	
Fecal total SCFAs, µmol/g	Median (IQR)	36.4 (24.9–45.8)	35 (25–40)	43 (23–55)	0.11
Reference value according to the lab	µmol/g	≥14	≥14	≥14	
Abnormal values	*n*/N (%)	2/60 (3)	0/40 (0)	2/20 (10)	
Fecal butyrate, µmol/g	Median (IQR)	4 (2.4–7.6)	3.4 (2.3–6.1)	4.5 (2.9–8.8)	0.34
Reference value according to the lab	µmol/g	≥2.5	≥2.5	≥2.5	
Abnormal values	*n*/N (%)	15/59 (25)	12/40 (30)	3/19 (16)	
Serum zonulin, ng/mL	Median (IQR)	32 (28–42)	32 (28–42)	33 (28–42)	1
Reference cut-off value	ng/mL	>34	>34	>34	
Abnormal values	*n*/N (%)	23/61 (38)	16/41 (39)	7/20 (35)	
Serum calprotectin, µg/mL	Median (IQR)	2 (1.3–2.9)	2 (1.3–2.9)	1.7 (1.2–2.8)	1
Reference value according to the lab	µg/mL	<3	<3	<3	
Abnormal values	*n*/N (%)	14/61 (23)	10/41 (24)	4/20 (20)	
Plasma LPS, ng/mL	Median (IQR)	0.3 (0.2–0.5)	0.36 (0.3–0.5)	0.33 (0–0.4)	**0.018**
Cut-off value	ng/mL	>0.05	>0.05	>0.05	
Abnormal values	*n*/N (%)	26/30 (87)	18/18 (100)	8/12 (67)	

SCFAs: short-chain fatty acids; LPS: lipopolysaccharide.

**Table 3:** Frequencies of abnormal values of biomarkers related to obesity and RA.

	Biomarker	N			*p*-Value
Obesity			**non-obese**	**obese**	
	Serum zonulin, ng/mL	61	36% (15/42)	42% (8/19)	0.78
	Serum calprotectin, µg/mL	61	26% (11/42)	16% (3/19)	0.52
	Fecal zonulin, ng/g	61	97.6% (41/42)	53% (10/19)	1
	Fecal calprotectin, µg/g	61	31% (13/42)	53% (10/19)	0.15
	Plasma LPS, ng/mL	30	95% (19/20)	70% (7/10)	0.095
Rheumatoid Factor			**positive**	**negative**	
	Serum zonulin, ng/mL	61	62% (13/21)	26% (10/39)	**0.011**
	Serum calprotectin, µg/mL	61	38% (8/21)	15% (6/39)	0.061
	Fecal zonulin, ng/g	61	100% (21/21)	97.4% (38/39)	1
	Fecal calprotectin, µg/g	61	24% (5/21)	46% (18/39)	0.1
	Plasma LPS, ng/mL	30	85% (11/13)	88% (15/17)	1
CRP			**≥0.2**	**<0.2**	
	Serum zonulin, ng/mL	61	25% (8/32)	52% (15/29)	**0.038**
	Serum calprotectin, µg/mL	61	22% (7(32)	24% (7/29)	1
	Fecal zonulin, ng/g	61	100% (32/32)	96.6% (28/29)	047
	Fecal calprotectin, µg/g	61	34% (11/32)	41% (12/29)	0.61
	Plasma LPS, ng/mL	30	88% (15/17)	85% (11/13)	1
Glucocorticoids Treatment			**yes**	**no**	
	Serum zonulin, ng/mL	61	35% (6/17)	39% (17/44)	1
	Serum calprotectin, µg/mL	61	35% (6/17)	18% (8/44)	0.18
	Fecal zonulin, ng/g	61	100% (17/17)	97.7% (43/44)	1
	Fecal calprotectin, µg/g	61	59% (10/17)	30% (13/44)	**0.044**
	Plasma LPS, ng/mL	30	100% (6/6)	83% (20/24)	0.56
TNF-Inhibitor Treatment			**yes**	**no**	
	Serum zonulin, ng/mL	61	36% (4/11)	38% (19/50)	1
	Serum calprotectin, µg/mL	61	27% (3/11)	22% (11/50)	0.7
	Fecal zonulin, ng/g	61	100% (11/11)	98% (49/50)	1
	Fecal calprotectin, µg/g	61	64% (7/11)	32% (16/50)	0.084
	Plasma LPS, ng/mL	30	66.7% (4/6)	91.7% (22/24)	0.17

**Table 4:** Univariate and multivariate linear regression models.

Dependent Variable	Independent Variable	Univariable Coefficient	*p*-Value	Multi- Variable Coefficient (R^2^)	*p*-Value
Serum zonulin (ng/mL)	Gender, m/f	1.03	0.718	−0.34	0.908
	Age, years	0.02	0.857	−0.00	0.999
	BMI, kg/m^2^	0.18	0.507	0.26	0.333
	SDAI, units	0.11	0.489	0.09	0.548
	Disease duration, years	0.29	**0.017**	0.29	**0.016**
	Fecal zonulin, ng/g	0.02	**0.049**	0.01	**0.047**
Serum calprotectin (µg/mL)	Gender, m/f	0.06	0.875	0.15	0.714
	Age, years	−0.02	0.223	−0.02	0.247
	BMI, kg/m^2^	−0.06	0.094	−0.07	0.061
	SDAI, units	0.02	0.381	0.01	0.725
	Fecal calprotectin, µg/g	0.00	**0.040**	0.00	**0.027**
Fecal zonulin (ng/g)	Gender, m/f	35.25	0.453	46.20	0.0339
	Age, years	−4.49	**0.028**	−5.28	**0.013**
	BMI, kg/m^2^	0.66	0.882	3.99	0.356
	SDAI, units	1.45	0.581	0.65	0.793
	Fecal total SCFAs, (µmol/g)	−0.51	0.095	4.17	0.119
	Fecal butyrate, (µmol/g)	−2.40	0.065	−19.29	0.088
	Serum zonulin, ng/mL	4.16	**0.049**	3.49	0.088
Fecal calprotectin (µg/g)	Gender, m/f	−15.15	0.650	−57.38	0.406
	Age, years	0.71	0.631	−0.09	0.977
	BMI, kg/m^2^	1.65	0.601	3.26	0.688
	SDAI, units	2.15	0.247	1.98	0.634
	Serum calprotectin, µg/mL	21.59	**0.040**	32.84	0.059
	Plasma LPS, ng/mL	163.54	0.074	196	**0.043**

Abbreviations: BMI, body mass index; SDAI, simple disease activity index; SCFAs, short-chain fatty acids; LPS, lipopolysaccharide; R^2^, multivariable coefficient of determination.

## Data Availability

Not applicable.

## Supplementary Materials

The following supporting information can be downloaded at https://www.mdpi.com/article/10.3390/nu15102386/s1. Figure S1—Linear regression: serum zonulin and serum calprotectin with LPS; Figure S2—Linear regression: Fecal zonulin and fecal calprotectin with LPS; Figure S3—Linear regression: Fecal zonulin with serum zonulin in males and females; Figure S4—Linear regression: serum calprotectin with LPS in males and females; Figure S5—Linear regression: serum zonulin wit LPS in males and females; Figure S6—Linear regression: fecal zonulin with LPS in males and females; Table S1—Smoking habits and obesity status; Table S2—Frequencies of abnormal values of fecal and blood biomarkers related to RA (non-significant results); Table S3—Univariate and multivariate linear regression models (non-significant results).
