# Supplementary material for: Assessment of Intestinal Permeability and Inflammation Bio-Markers in Patients with Rheumatoid Arthritis
Source: Nutrients. 2023 May 19;15(10):2386. doi: 10.3390/nu15102386 (PMC10221762; doi:10.3390/nu15102386)
Supplement: Supplementary file 1 [file nutrients-15-02386-s001.zip › Table S1 Smoking habits and obesity status.pdf]

Pat-ID			Smoking status			Comorbidity
	Gender	Age	Current	Former	Never	Obesity
1	f	54	0	0	1	0
2	f	67	0	1	0	0
3	f	69	0	0	1	0
4	f	71	0	1	0	0
5	f	61	0	0	1	1
6	f	65	0	1	0	0
7	m	59	0	0	1	0
8	m	56	0	1	0	0
9	f	71	0	0	1	0
10	f	57	0	0	1	0
11	f	79	0	0	1	1
12	f	66	1	0	0	0
13	m	48	0	0	1	0
14	m	57	0	1	0	0
15	f	74	0	0	1	1
16	f	73	0	0	1	0
17	m	51	0	1	0	0
18	m	70	1	0	0	0
19	f	73	0	1	0	0
20	f	56	0	1	0	1
21	f	64	0	1	0	0
22	f	74	0	1	0	1
23	f	69	0	1	0	0
24	m	55	0	1	0	1
25	m	70	0	1	0	0
26	m	45	1	0	0	1
27	m	43	0	0	1	0
28	f	56	0	1	0	1
29	f	62	0	0	1	1
30	f	31	0	0	1	0
31	f	66	0	1	0	0
32	m	78	0	0	1	0
33	f	63	0	0	1	1
34	m	45	0	0	1	1
35	m	58	0	1	0	1
36	f	62	0	0	1	0
37	m	57	1	0	0	0
38	m	57	0	0	1	0
39	f	60	1	0	0	0
40	f	65	0	0	1	0
41	f	31	0	0	1	0
42	m	54	0	1	0	0
43	f	65	0	1	0	0
44	f	72	0	0	1	0
45	m	66	0	1	0	1
46	m	73	0	1	0	0
47	f	63	0	1	0	0

48	f	55	0	1	0	1
49	m	58	0	0	1	0
50	f	73	1	0	0	1
51	f	58	0	1	0	0
52	f	45	0	1	0	0
53	f	66	0	1	0	0
54	f	70	0	0	1	1
55	f	57	0	0	1	0
56	m	60	0	1	0	1
57	f	78	0	0	1	1
58	f	80	0	0	1	0
59	f	53	0	0	1	0
60	f	71	0	0	1	1
61	f	74	0	0	1	0
