# Supplementary material for: Assessment of Intestinal Permeability and Inflammation Bio-Markers in Patients with Rheumatoid Arthritis
Source: Nutrients. 2023 May 19;15(10):2386. doi: 10.3390/nu15102386 (PMC10221762; doi:10.3390/nu15102386)
Supplement: Supplementary file 1 [file nutrients-15-02386-s001.zip › Table S2 Frequencies of abnormal values of fecal and blood biomarkers (non-significant results).pdf]

Table S2: Frequencies of abnormal values of fecal and blood biomarkers (non-significant results)

A: related to SDAI
B: related to disease duration
C: Smoking status
D: BMI, ACPA, drug treatment

Table S2 A: Frequency of abnormal values of fecal and blood biomarkers related to SDAI.

SDAI, units	N	0.0 - 3.3	3.4 - 11.0	11.1 - 26.0	26.1 - 86.0	p-value
		remission	low activity	moderate activity	high activity	
Serum Zonulin, ng/ml	61	43% (3/7)	35% (8/23)	42% (11/26)	20% (1/5)	0.88
Serum Calprotectin, µg/ml	61	29% (2/7)	22% (5/23)	15% (4/26)	60% (3/5)	0.17
Fecal Zonulin, ng/g	61	100% (7/7)	95.7% (22/23)	100% (26/26)	100% (5/5)	0.57
Fecal Calprotectin, µg/g	61	0% (0/7)	39% (9/23)	46% (12/26)	40% (2/5)	0.15
Plasma LPS, ng/ml	30	80% (4/5)	77% (10/13)	100% (10/10)	100% 2/2)	0.41

Table S2 B: Frequency of abnormal values of fecal and blood biomarkers related to disease duration.

Disease duration, years	N	0-2	3-10	> 10	p-value
Serum Zonulin, ng/ml	61	29% (8/28)	40% (8/20)	54% (7/13)	0.25
Serum Calprotectin, µg/ml	61	29% (8/28)	20% (4/20)	15% (2/13)	0.73
Fecal Zonulin, ng/g	61	100% (28/28)	95% (19/20)	100% (13/13)	0.54
Fecal Calprotectin, µg/g	61	36% 10/28)	45% (9/20)	31% (4/13)	0.75
Plasma LPS, ng/ml	30	80% (8/10)	90.9% (10/11)	88.9% (8/9)	0.83

Table S2 C: Frequency of abnormal values of fecal and blood biomarkers related to smoking status.

Smoking Status	N	Current	Former	Never	p-value
Serum Zonulin, ng/ml	61	33% (2/6)	38% (10/26)	38% (11/29)	1
Serum Calprotectin, µg/ml	61	17% (1/6)	19% (5/26)	28% (8/29)	0.82
Fecal Zonulin, ng/g	61	100% (6/6)	96.2% (25/26)	100% (29/29)	0.52
Fecal Calprotectin, µg/g	61	50% (3/6)	31% (8/26)	41% (12/29)	0.64
Plasma LPS, ng/ml	30	66.7% (2/3)	91.7% (11/12)	86.7% (13/15)	0.55

Table S2 D: Frequency of abnormal values of fecal and blood biomarkers related to smoking BMI, ACPA, and drug treatment.

	Biomarker	N			p-value
			≥ 25	< 25	
BMI	Serum Zonulin, ng/ml	61	32% (12/37)	46% (11/24)	0.42
	Serum Calprotectin, µg/ml	61	16% (6/37)	33% (8/24)	0.14
	Fecal Zonulin, ng/g	61	97.3% (36/37)	100% (24/24)	1
	Fecal Calprotectin, µg/g	61	46% (17/37)	25% (6/24)	0.15
	Plasma LPS, ng/ml	30	82% (18/22)	100% (8/8)	0.55
			**positive**	**negative**	
ACPA	Serum Zonulin, ng/ml	61	50% (8/16)	34% (15/44)	0.37
	Serum Calprotectin, µg/ml	61	31% (5/16)	20% (9/44)	0.49
	Fecal Zonulin, ng/g	61	100% (16/16)	97.7% (43/44)	1

	Fecal Calprotectin, µg/g	61	31% (5/16)	41% (18/44)	0.56
	Plasma LPS, ng/ml	30	89% (8/9)	86% (18/21)	1
			**yes**	**no**	
Methotrexate (MTX)	Serum Zonulin, ng/ml	61	43% (9/21)	35% (14/40)	0.59
	Serum Calprotectin, µg/ml	61	33% (7/21)	18% (7/40)	0.21
	Fecal Zonulin, ng/g	61	100% (21/21)	97.5% (39/40)	1
	Fecal Calprotectin, µg/g	61	38% (8/21)	38% (15/40)	1
	Plasma LPS, ng/ml	30	90% (9/10)	85% (17/20)	1
			**yes**	**no**	
MTX + Glucocorticoids	Serum Zonulin, ng/ml	61	60% (3/5)	39% (11/28)	0.52
	Serum Calprotectin, µg/ml	61	60% (3/)	14% (4/28)	0.1
	Fecal Zonulin, ng/g	61	100% (5/5)	96.% (27/28)	1
	Fecal Calprotectin, µg/g	61	60% (3/5)	29% (8/28)	0.31
	Plasma LPS, ng/ml	30	100% (2/2)	81.2% (13/16)	0.56
			**yes**	**no**	
Leflunomide/ Sulfasalazine Treatment	Serum Zonulin, ng/ml	61	33% (3/9)	39% (20/51)	1
	Serum Calprotectin, µg/ml	61	11% (1/9)	26% (13/51)	0.67
	Fecal Zonulin, ng/g	61	100% (9/9)	98% (50/51)	1
	Fecal Calprotectin, µg/g	61	22% (2/9)	39% (20/51)	0.46
	Plasma LPS, ng/ml	30	100% (5/5)	84% (21/25)	1
			**yes**	**no**	
Leflunomide/Sulfa salazine + Glucocorticoids Treatment	Serum Zonulin, ng/ml	61	50% (2/4)	41% (16/39)	0.61
	Serum Calprotectin, µg/ml	61	25% (1/4)	20% (8/39)	0.79
	Fecal Zonulin, ng/g	61	100% (4/4)	97.4% (38/39)	1
	Fecal Calprotectin, µg/g	61	25% (1/4)	31% (12/39)	0.3
	Plasma LPS, ng/ml	30	100% (2/2)	81% (17/21)	0.69
			**yes**	**no**	
JAK-Inhibitor Treatment	Serum Zonulin, ng/ml	61	33% (1/3)	38% (22/58)	1
	Serum Calprotectin, µg/ml	61	33% (1/3)	22% (13/58)	0.55
	Fecal Zonulin, ng/g	61	100% (3/3)	98.3% (57/58)	1
	Fecal Calprotectin, µg/g	61	33% (1/3)	38% (22/58)	1
	Plasma LPS, ng/ml	30	100% (3/3)	85% (23/27)	1
