# Supplementary material for: Assessment of Intestinal Permeability and Inflammation Bio-Markers in Patients with Rheumatoid Arthritis
Source: Nutrients. 2023 May 19;15(10):2386. doi: 10.3390/nu15102386 (PMC10221762; doi:10.3390/nu15102386)
Supplement: Supplementary file 1 [file nutrients-15-02386-s001.zip › Table S3 Univariate and multivariate linear regression models .pdf]

Table S3: Univariate and multivariate linear regression models (non-significant results)

Dependent Variable	Independent Variable	Univariable Coefficient	p-value	Multi-variable Coefficient (R^2)	p-value
Serum Zonulin (ng/ml)	CRP, mg/dL	2.62	0.118		
	Total Cholesterol, mmol/L	0.00	0.893		
	Non-HDL-Cholesterol, mmol/L	0.01	0.721		
	Fasting Triglycerides, mmol/L	0.03	0.206		
	Total Fiber Intake, g	0.02	0.926		
	Fecal Total SCFAs, (μmol/g)	-0.00	0.947		
	Fecal Butyrate, (μmol/g)	-0.01	0.863		
	Plasma LPS, ng/ml	6.23	0.384		
Serum Calprotectin (μg/ml)	Disease duration, years	0.01	0.591		
	CRP, mg/dL	0.25	0.294		
	Total Cholesterol, mmol/L	-0.01	0.192		
	Non-HDL-Cholesterol, mmol/L	-0.01	0.253		
	Fasting Triglycerides, mmol/L	0.00	0.716		
	Total Fiber Intake, g	0.01	0.639		
	Fecal Total SCFAs, (μmol/g)	0.00	0.944		
	Fecal Butyrate, (μmol/g)	0.00	0.924		
	Plasma LPS, ng/ml	-0.23	0.825		
Fecal Zonulin (ng/g)	Disease duration, years	0.38	0.853		
	CRP, mg/dL	-10.72	0.701		
	Total Cholesterol, mmol/L	0.30	0.555		
	Non-HDL-Cholesterol, mmol/L	0.31	0.547		
	Fasting Triglycerides, mmol/L	0.25	0.451		
	Total Fiber Intake, g	-0.50	0.873		
	Plasma LPS, ng/ml	-8.66	0.937		

Fecal Calprotectin (μg/g)	Disease duration, years	-0.97	0.508		
	CRP, mg/dL	6.45	0.745		
	Total Cholesterol, mmol/L	-0.14	0.707		
	Non-HDL-Cholesterol, mmol/L	-0.18	0.623		
	Fasting Triglycerides, mmol/L	-0.29	0.223		
	Total Fiber Intake, g	-0.55	0.807		
	Fecal Total SCFAs, (μmol/g)	-0.07	0.738		
	Fecal Butyrate, (μmol/g)	-0.29	0.762		
