# Supplementary figures and images for: Assessment of Intestinal Permeability and Inflammation Bio-Markers in Patients with Rheumatoid Arthritis
Source: Nutrients. 2023 May 19;15(10):2386. doi: 10.3390/nu15102386 (PMC10221762; doi:10.3390/nu15102386)

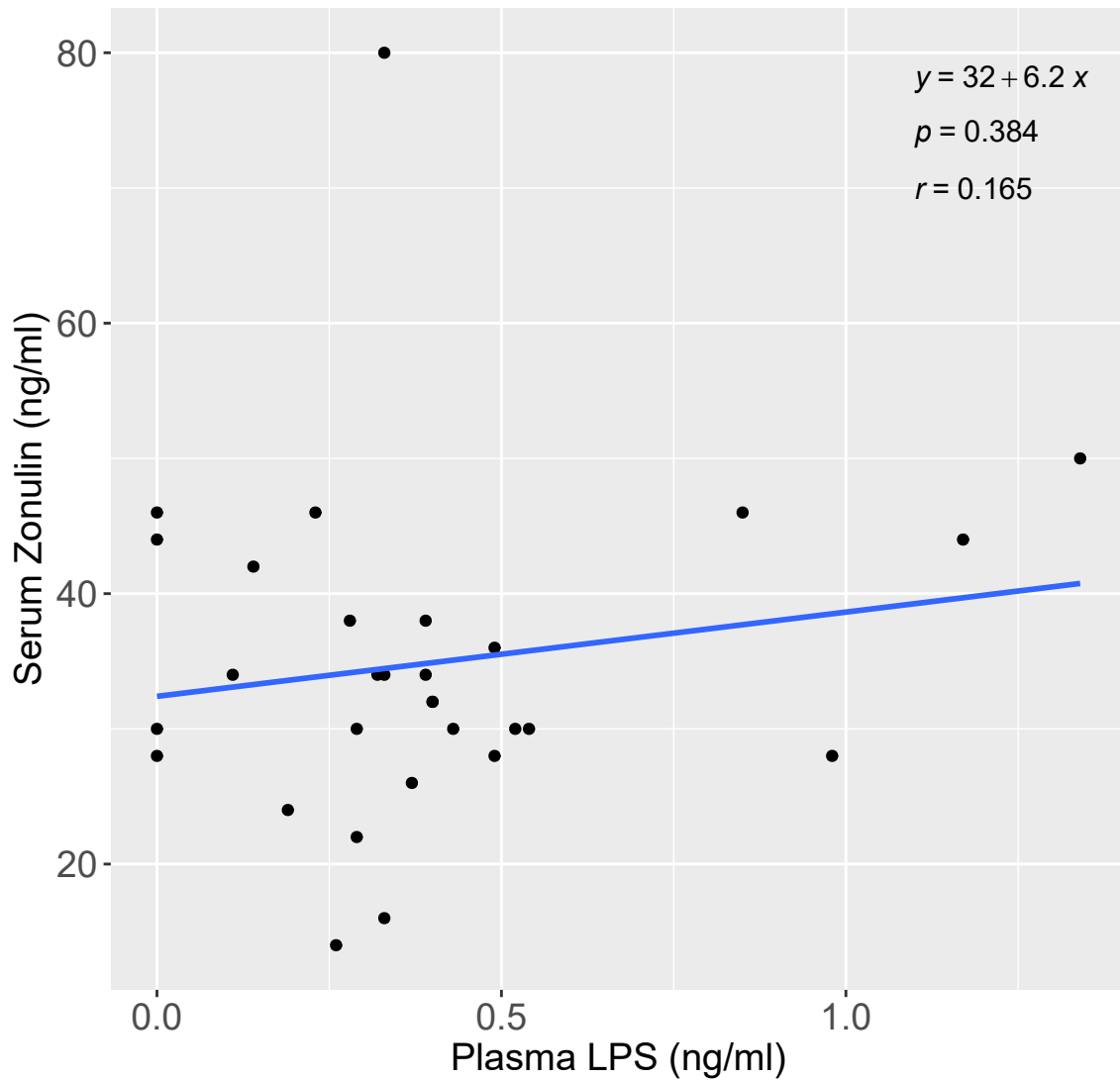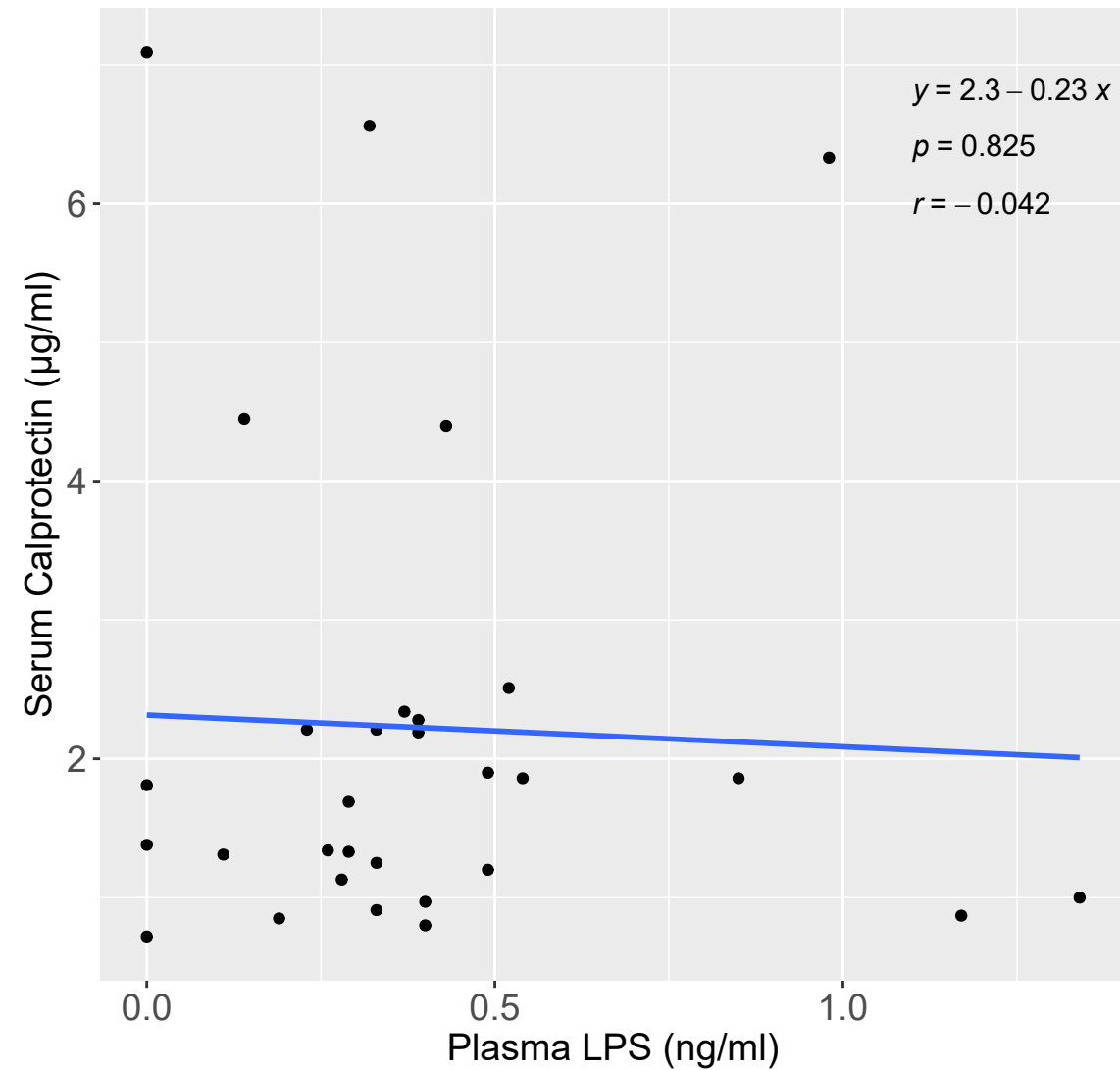

Supplement: Supplementary file 1 [file nutrients-15-02386-s001.zip › Figure S1 Serum Zonulin and Serum Calprotectin with Plasma LPS.PDF]

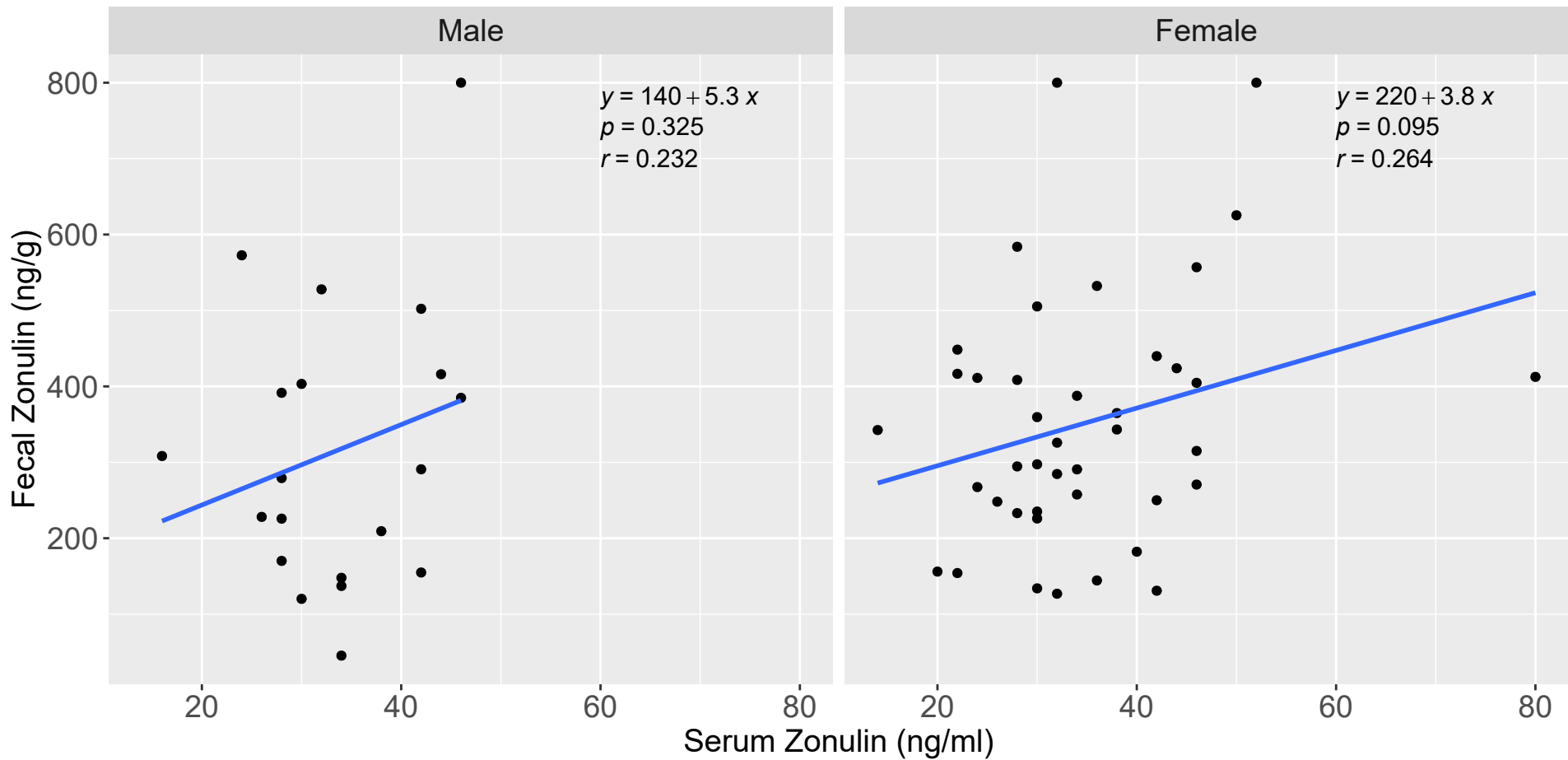

Supplement: Supplementary file 1 [file nutrients-15-02386-s001.zip › Figure S3 Fecal Zonulin and Serum Zonulin in males and females.PDF]

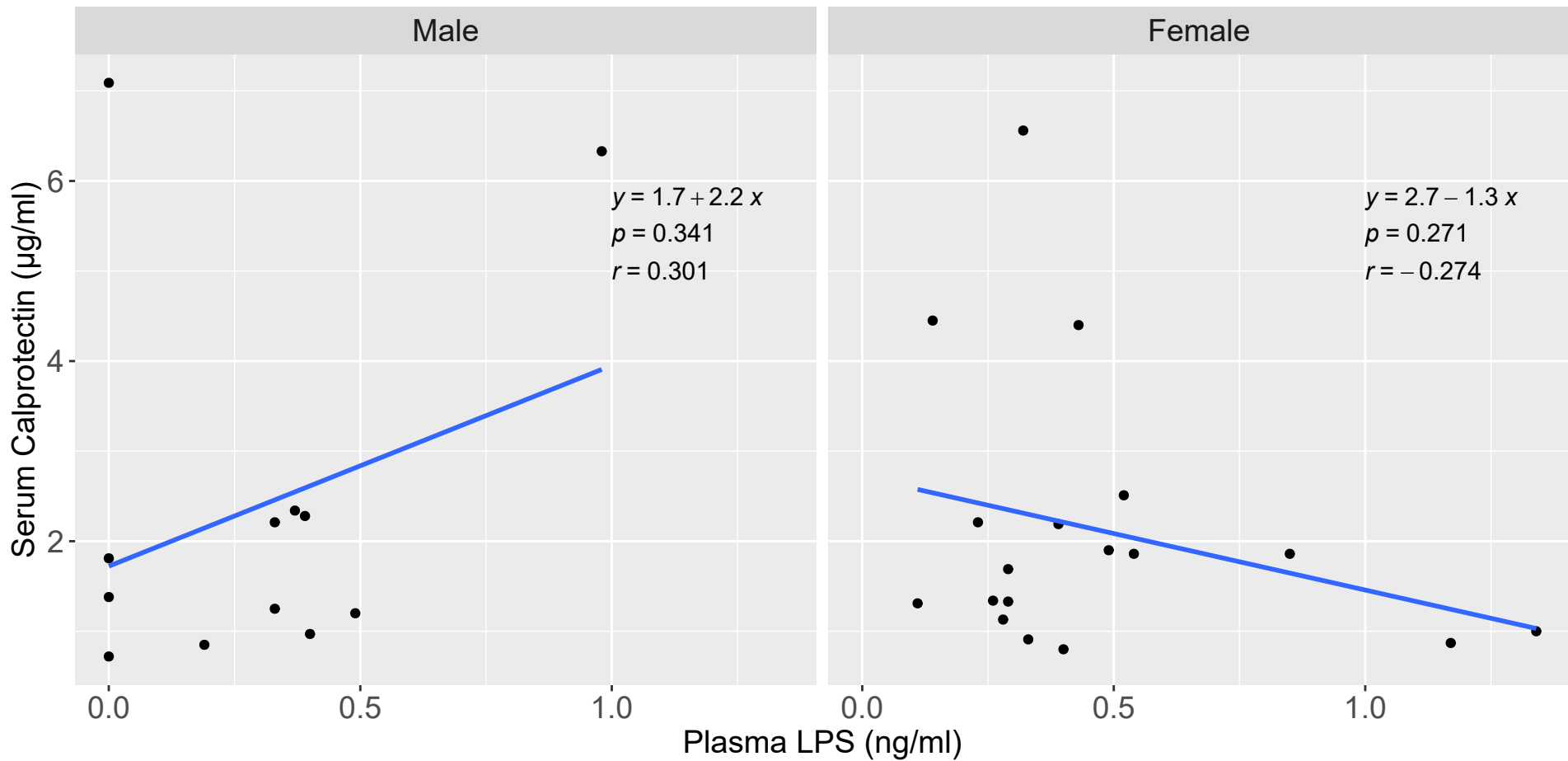

Supplement: Supplementary file 1 [file nutrients-15-02386-s001.zip › Figure S4 Serum Calprotectin and Plasma LPS in males and females.PDF]

Serum Zonulin (ng/ml)

Male

$y = 34 - 11x$   
 $p = 0.243$   
 $r = -0.366$

0.0

0.5

1.0

Plasma LPS (ng/ml)

Female

$y = 33 + 10x$   
 $p = 0.315$   
 $r = 0.251$

0.0

0.5

1.0

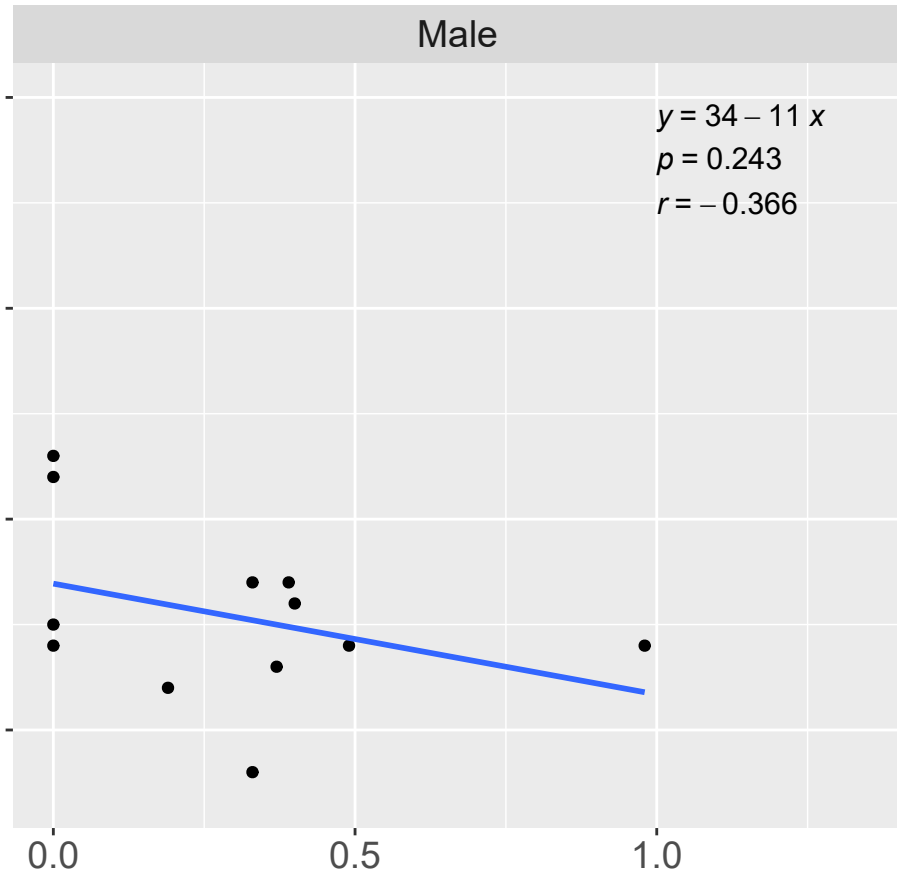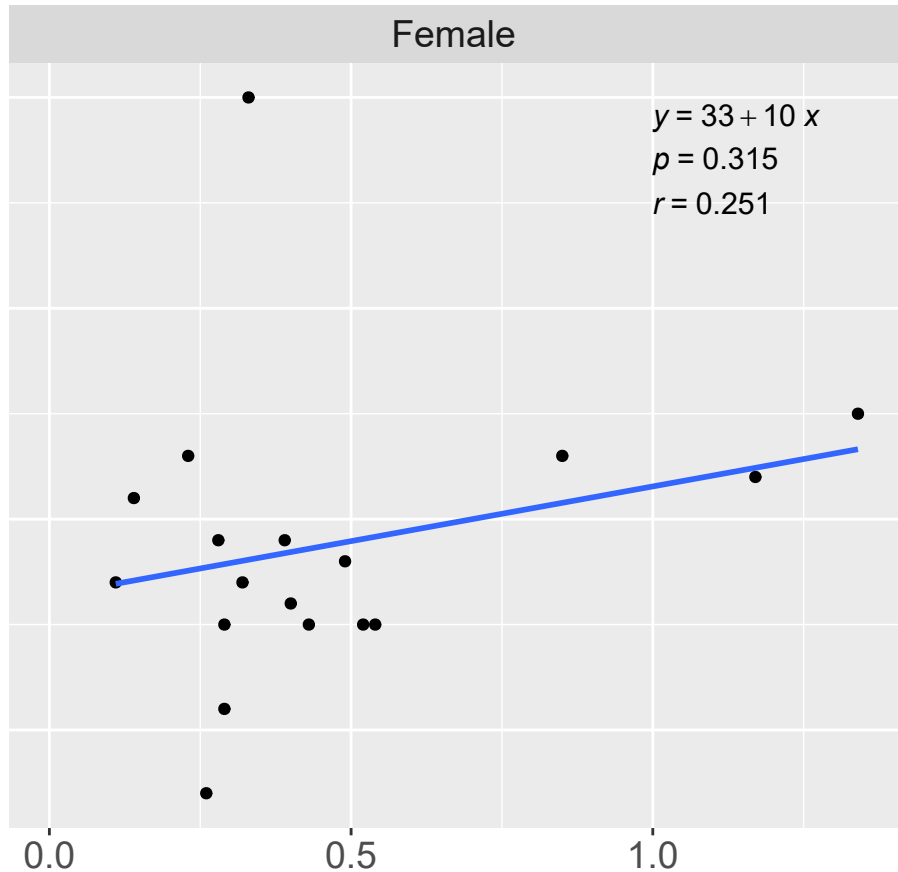

Supplement: Supplementary file 1 [file nutrients-15-02386-s001.zip › Figure S5 Serum Zonulin and Plasma LPS in males and females.PDF]

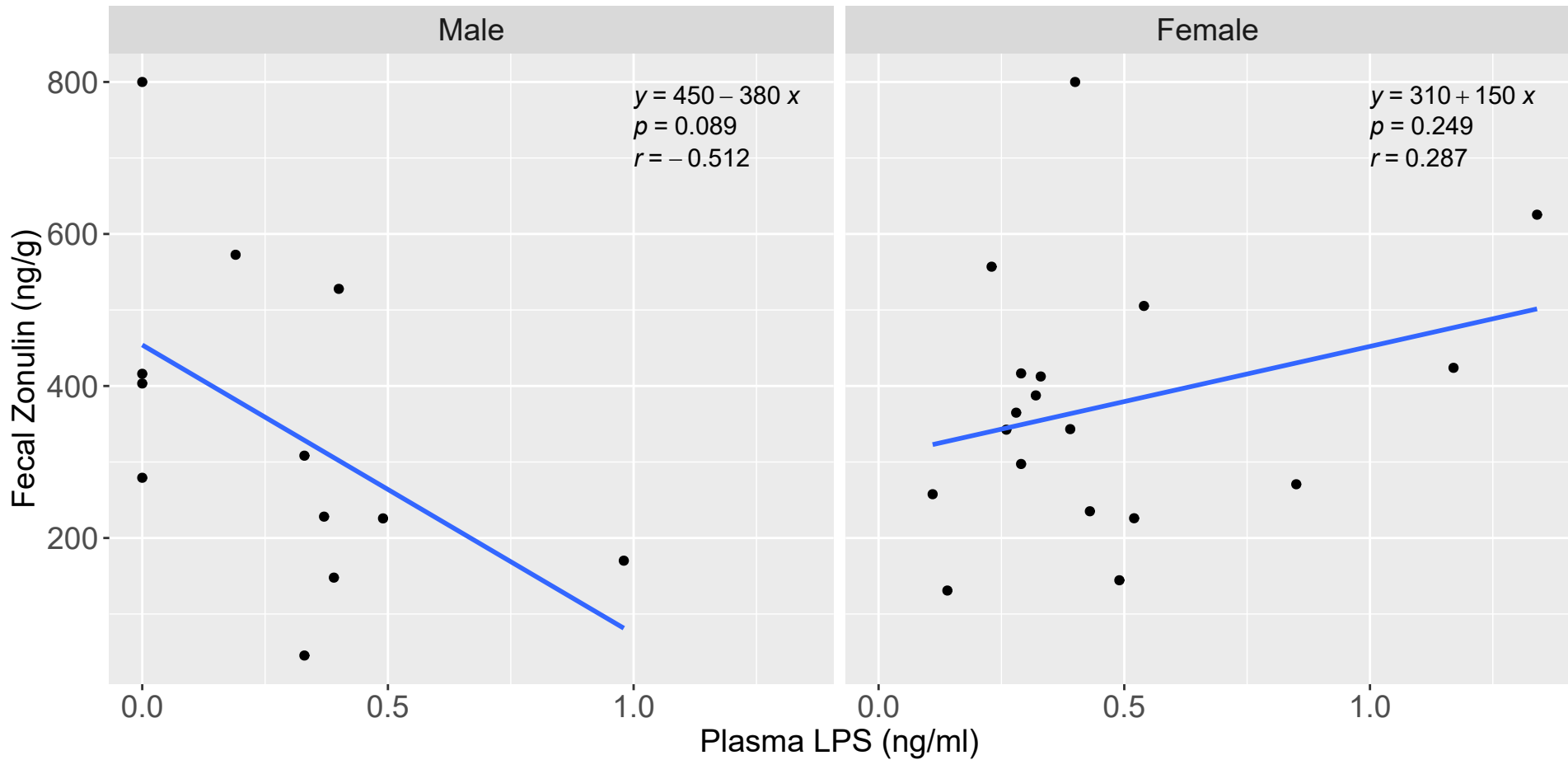

Supplement: Supplementary file 1 [file nutrients-15-02386-s001.zip › Figure S6 Fecal Zonulin and Plasma LPS in males and females.PDF]
