# Supplementary material for: Assessment of Intestinal Permeability and Inflammation Bio-Markers in Patients with Rheumatoid Arthritis
Source: Nutrients. 2023 May 19;15(10):2386. doi: 10.3390/nu15102386 (PMC10221762; doi:10.3390/nu15102386)
Supplement: Supplementary file 1 [file Figure S2 Fecal Zonulin and Fecal Calprotectin with Plasma LPS.PDF › Figure S2 Fecal Zonulin and Fecal Calprotectin with Plasma LPS.PDF]

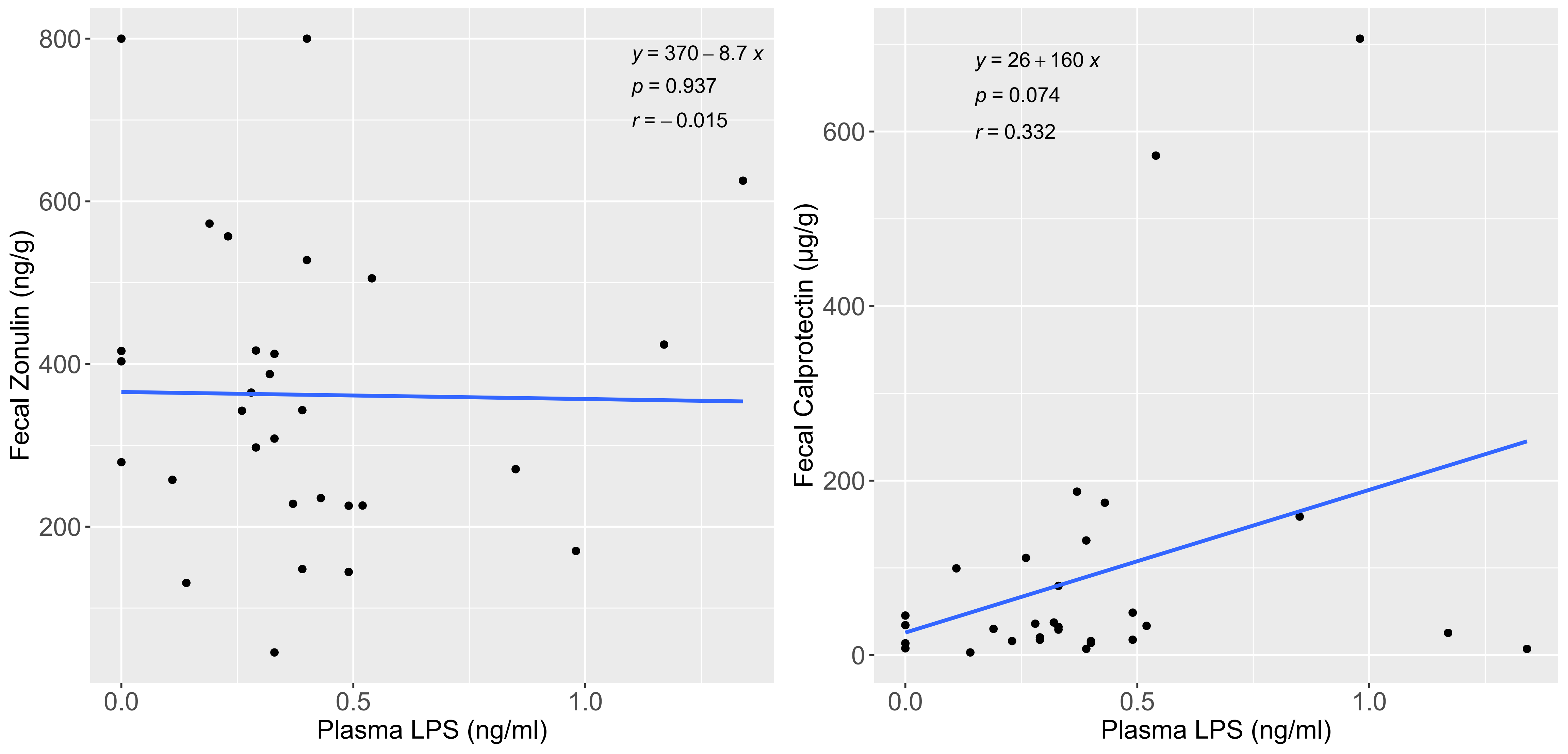

y = 370 – 8.7 x
p = 0.937
r = – 0.015
Fecal Zonulin (ng/g)
800
600
400
200
0.0
0.5
1.0
Plasma LPS (ng/ml)
y = 26 + 160 x
p = 0.074
r = 0.332
Fecal Calprotectin (µg/g)
600
400
200
0
0.0
0.5
1.0
Plasma LPS (ng/ml)
